# Time of day dependent longitudinal changes in resting-state fMRI

**DOI:** 10.3389/fneur.2023.1166200

**Published:** 2023-07-05

**Authors:** Liucija Vaisvilaite, Micael Andersson, Alireza Salami, Karsten Specht

**Affiliations:** ^1^ReState Research Group, Department of Biological and Medical Psychology, University of Bergen, Bergen, Norway; ^2^Mohn Medical and Imaging Visualization Centre, Haukel and University Hospital, Bergen, Norway; ^3^Umeå Center for Functional Brain Imaging, Umeå University, Umeå, Sweden; ^4^Department of Integrative Medical Biology, Umeå University, Umeå, Sweden; ^5^Ageing Research Center, Karolinska Institute, Stockholm, Sweden; ^6^Department of Education, UiT/The Arctic University of Norway, Tromsø, Norway

**Keywords:** resting-state, fMRI, dynamic causal modeling (DCM), time of day (ToD), circadian rythm

## Abstract

Longitudinal studies have become more common in the past years due to their superiority over cross-sectional samples. In light of the ongoing replication crisis, the factors that may introduce variability in resting-state networks have been widely debated. This publication aimed to address the potential sources of variability, namely, time of day, sex, and age, in longitudinal studies within individual resting-state fMRI data. DCM was used to analyze the fMRI time series, extracting EC connectivity measures and parameters that define the BOLD signal. In addition, a two-way ANOVA was used to assess the change in EC and parameters that define the BOLD signal between data collection waves. The results indicate that time of day and gender have significant model evidence for the parameters that define the BOLD signal but not EC. From the ANOVA analysis, findings indicate that there was a significant change in the two nodes of the DMN and their connections with the fronto-parietal network. Overall, these findings suggest that in addition to age and gender, which are commonly accounted for in the fMRI data collection, studies should note the time of day, possibly treating it as a covariate in longitudinal samples.

## 1. Introduction

Resting-state functional magnetic resonance imaging (fMRI) has been exponentially used in both research and clinical studies (1). The resting-state paradigm has become a part of standard fMRI acquisition protocols and has been included in large-scale neuroimaging studies such as the Human Connectome Project, UK Biobank, and ABCD study (2–4). Many of the large-scale data initiatives as the examples mentioned in addition to investigating the neural correlates of behavior, cognition, and mental health in part want to address the issues of reliability, that have been brought up during the recent years (5). It is broadly agreed that there are many factors that may funnel into the so-called replication crisis in neuroimaging (6). It is stressed that there is a pressing need for more open protocols, data and code sharing, clear communication, and rigorous and comparable procedures across studies (7, 8).

Up until quite recently, the majority of resting-state fMRI studies relied on cross-sectional samples even though it is noted that longitudinal data allowing for within-individual comparison is by far superior (9). As a result, the majority of large-scale data collection and consortium initiatives involved repeated measures, which are collected on multiple occasions not only for the neuroimaging samples but also health, cognitive, and neuropsychological testing (2–4). The longitudinal data allows for addressing developmental changes in more detail and has been extremely informative for studies investigating both healthy or pathological aging and child development (10, 11). One example of such data collection initiatives is the BETULA, a longitudinal study on aging, memory, and dementia (12), which is an ongoing longitudinal study on aging, memory, and dementia in Umeå, Sweden.

Analyzing longitudinal data allows for tracking individual changes over time, where time usually spans from days and months to years. The changes observed are then conventionally attributed to aging, development, pathology progression, or similar factors. For example, a study by Oschmann and Gawryluk (11) looked at longitudinal data between network connectivity changes in pathology-free aging, where they report a decline in functional connectivity (FC) in the fronto-parietal network and saliency network (SN), with stable connectivity for the default mode network (DMN) (11).

However, there may be additional factors that may introduce change or variability in the data, that are rarely accounted for in the data acquisition protocols (13). A previously published study indicates that time of day (TOD), i.e., time of the image acquisition, influences the blood oxygen level-dependent (BOLD) signal throughout the day (14). In addition, it has been shown that the low-frequency fluctuations vary based on the time of the fMRI acquisition (15). It is important to note that all the referenced studies relied on between-individuals cross-sectional designs, and the results highlighted are based on between-group comparisons.

The diurnal changes are not limited to functional studies. Research using structural MRI reports that there were significant time of day-dependent local gray and white matter changes, which can be attributed to circadian-related processes (16). These findings are supported by others, comparing morning and evening scans of healthy volunteers (17). In addition, a large longitudinal comparison of clinical populations yields similar findings—time of day-dependent volume changes in individuals with dementia (18).

However, the assumption of circadian rhythms' involvement in brain dynamics is challenged by Fafrowicz et al. (15). The authors report that individual circadian preference (early vs. late phenotype) did not yield any significant difference in the resting-state network organization. While these are somewhat theoretically conflicting findings, they are not in conflict with the time of image acquisition-dependent changes but rather stress that the individual circadian preference does not play a significant role. From this and earlier studies, a common conclusion can be drawn—time of day as meant in the sense of time of image acquisition seems to be a factor that influences the organization and morphological metrics of the human brain.

To date, only a handful of studies investigated the circadian mechanisms and time of image acquisition in within-individual samples of healthy volunteers. A study by Shannon et al. (19) has indicated that the functional brain organization is not static over the course of the day (19) while using within-individual design, a comparison was made between evening and morning data. The publication “time of day matters” similarly compared individual scans acquired during the morning session and evening session (16). Finally, one recent study investigated the physiological factors, such as blood pressure, circadian rhythms, hydration, and caffeine levels in healthy controls over 3 weeks (20). While the authors of the mentioned study report minimal time of day effects, they note that the difference between scanning occasions was short, which may be the reason for the lack of significant effects of time of day. It is important to note that many of the studies mentioned relied on repeated measure design rather than longitudinal data *per se*, as the time between data collection occasions in the span of days, aside from the study by Zahid et al. (20). Therefore, given the vastly different temporal aspects, these results cannot be straightforwardly generalized to longitudinal samples, where data collection waves may span from months to years.

The current publication, therefore, addresses the existing gap in the academic literature. The study focuses on additional factors, namely time of day, sex, and years of age changes in a longitudinal sample, with a specific focus on the consistency of the image acquisition time per individual. We believe that maintaining the same acquisition time span may remove the added variability in effective connectivity (EC) and BOLD signal measures, which, if kept, would allow for a more precise track of differences over time in longitudinal samples, especially studies addressing aging.

## 2. Materials and methods

### 2.1. Participants

The data used in the present study have been collected as a part of the BETULA project on memory, health, and aging (12). The BETULA study is a currently ongoing longitudinal project at Umeå University, Umeå, Sweden. To date, there have been a total of seven data collection occasions [time point of collection (T)]; for the purposes of the current publication, the data from collection waves T5 and T6 were used. The data collection waves T5 and T6 were done approximately 4 years apart, specifically T5 during 2009–2010 and T6 during 2013–2014. The data collection, selection, and further processing descriptions in the Methods section will only refer to these particular data collection occasions unless explicitly otherwise stated.

Structural and functional MRI scans were collected from a total of 375 participants during the T5 data collection wave. A total of 37 scans were excluded from the total samples due to technical error and/or severe motion within the scanner. In addition, 84 participants had to be excluded because of pre-existing neuropsychological illness and/or dementia (21). Finally, 56 participant scans were removed after the data were quality checked for mean framewise displacement (FD) in the scanner, with a conservative threshold of FD < 0.2 (22). The final sample of neuropsychologically healthy and quality-checked participants was 236.

For the data collection wave T6, a total of 301 participant data were available. After the quality check, 44 participant data were removed based on the selected FD threshold of FD < 0.2 (22). In addition, data from 55 participants were excluded based on the diagnosis of neuropsychological illness and/or dementia (21).

Given the aims of the current study, the additional sorting of the participant data was done as follows:

Only subjects that participated in both data collection waves T5 and T6 were selected. These were subsequently allocated into four groups based on image acquisition time and test wave. The final groups were as follows:

- Congruent time (morning group) scanned in the morning sessions from 7:30 to 9:30 during both t5 and t6
- Congruent time (afternoon group) scanned in the afternoon sessions from 14:00 to 18:00 during both t5 and t6
- Incongruent time (morning–afternoon) (swapped times of image acquisition) participants scanned in the morning session on t5 and afternoon session on t6
- Incongruent time (afternoon–morning) (swapped times of image acquisition) participants scanned in the afternoon session on t5 and morning session on t6

Complete information about the participant gender, age, and group sizes (N = total number of participants and *F* = number of females) is summarized in Table 1.

**Table 1:** Group sizes and participant information.

**Group**	**N (F)**	**Age at T5**	**Age at T6**
Congruent time (morning group)	25 (*F =* 11)	36–77	40–81
Congruent time (afternoon group)	11 (*F =* 5)	26–75	30–80
Incongruent time (morning–afternoon)	24 (*F =* 8)	25–70	29–74
Incongruent time (afternoon–morning)	13 (*F =* 3)	30–79	34–83

### 2.2. Data acquisition

A 3-T General Electric scanner equipped with a 32-channel head coil was used to acquire the scans. For the resting-state fMRI gradient, the echo-planar imaging sequence was used with the following specifications: 37 transaxial slices, thickness; 3.4 mm, gap; 0.5 mm, repetition time (TR); 2000 ms, echo time (TE); 30 ms, flip angle; 80°, field of view; 25 × 25 cm, 170 volumes. A total of 10 dummy scans were collected and then discarded before the experimental procedure. A 3D fast spoiled gradient-echo sequence was used to collect high-resolution T1-weighted structural images, with the following specifications: 180 slices; thickness, 1 mm; TR, 8.2 ms; TE, 3.2 ms; flip angle, 12°; field of view, 25 × 25 cm. The pCASL sequence was set using a stack of spiral fast spin-echo read-out, with 512 sampling points on eight spirals, FOV, 240 × 240 mm^2^; slice thickness 4 mm; arterial labeling at the cerebellar base; and 1500 ms labeling duration, 1525 ms post labeling delay, and 30 control/label pairs. Cerebral blood flow (CBF) maps were computed, showing tissue CBF in ml/min/100 g (12).

During the MRI session, the participants were instructed to lay as still as possible with their eyes open, looking at a white fixation cross on the dark screen.

### 2.3. Preprocessing

The Statistical Parametric Mapping software (SPM12; Welcome Department of Cognitive Neurology, University College London, London, United Kingdom) was used to preprocess the data (http://store.elsevier.com/product.jsp?isbn=9780123725608). The pre-processing steps are listed in the order they have been performed: slice-timing correction, realign, and un-warp; co-registration of the structure with the functional scans; segmentation of the structural scan; normalization of the functional scan; and, finally, smoothing of the data. The temporal middle slice (number 2 in an interleaved slice acquisition) was used as the reference image for the slice-timing correction. The data were then realigned and un-warped. For the co-registration of functional scans with the structural T1 images, the estimated mean images across the time series were used. The co-registered structural images were segmented for the gray matter, white matter, and cerebrospinal fluid. Thereafter, all functional volumes were normalized into MNI space using the segmented deformation fields. Finally, data were smoothed with a Gaussian kernel of 8 mm.

In addition to the standard pre-processing pipeline, the data were further processed for the DCM analysis. The packages implemented in SPM12 were used. A general linear model was used to regress out 12 head movement parameters together with the signals from the white matter and cerebrospinal fluid areas from the fMRI time series for each individual. In order to do so, the time course from a spherical volume with a radius of 6 mm at MNI coordinates (0-24-10) and (0-40-5), was extracted. Thereafter, these time courses and the movement parameters were incorporated into a general linear model. This then formed the basis for extracting the time courses for the following DCM analyses.

### 2.4. ROI selection

In total, 10 regions of interest (ROIs) were selected for a spectral DCM analysis. The coordinates for DMN, fronto-parietal, and SN networks were selected based on previous literature (23–25). The coordinates and anatomical locations for all ROIs are displayed in Table 2.

**Table 2:** MNI coordinates for selected ROIs.

	**x**	**y**	**z**	**Anatomical location**
SN	44	36	20	Dorsolateral prefrontal cortex (DLPFC)
SN	38	26	−10	Orbital frontoinsula (FI)
DMN	3	54	−2	Medial prefrontal cortex (mPFC)
DMN	0	−52	26	Posterior cingulate cortex (PCC)
DMN	48	−69	35	Right inferior parietal cortex (RIPC)
DMN	−50	−63	32	left inferior parietal cortex (LIPC)
Fronto-parietal	−52	−49	47	Left anterior parietal
Fronto-parietal	52	−46	46	Right anterior parietal
Fronto-parietal	−36	57	9	Left anterior prefrontal cortex (PFC)
Fronto-parietal	34	52	10	Right anterior prefrontal cortex (PFC)

### 2.5. Analysis

#### 2.5.1. Dynamic causal modeling

The effective connectivity and parameters defining the blood oxygen level-dependent signal (BOLD) signal, *epsilon*, d*ecay*, and *transit*, were estimated using spectral dynamic causal modeling (DCM). This was done for data items, i.e., per individual per each data collection wave.

#### 2.5.2. Parametric empirical bayes

The Parametric Empirical Bayes (PEB) framework was used to create a design matrix modeling repeated measures, i.e., two data items per each individual. In addition, four separate groups based on the participant sorting were specified, i.e., congruent morning, congruent afternoon, incongruent morning–afternoon, and incongruent afternoon–morning. Finally, the joint effective connectivity and BOLD parameters defining the signal were estimated per specified group (26, 27).

#### 2.5.3. Bayesian model comparison

Bayesian Model Comparison (BMC) was used to estimate the model fit to the group data. Model fit to the data, in detail, means that predefined variable or combination of variables values vary in synchrony with the fMRI time series. All the predefined models are summarized in Table 3. There were eight models in total. Briefly, they included all the variables addressed, namely, age, sex, and time of day with all the combinations of the three as well as the null model. All the models with the best fit were explored at a cutoff of the posterior probability (pp) >0.95.

**Table 3:** List of models specified for PEB analysis.

1. Time of image acquisition
2.Gender
3. Age
4. Time of image acquisition and sex
5. Time of image acquisition and age
6. Sex and age
7. Time of image acquisition, sex, and age
8. Null model: expectation that none of the models explain the variability in the data

The BMC and posterior probability estimation were conducted for the effective connectivity matrix (A-matrix, 10 × 10 parameter). The same procedure was repeated for the parameters defining the BOLD signal of the Balloon model, specifically *transit time, epsilon, decay*, and parameters α, which represented the amplitude and β, which reflected the spectral density of the neural fluctuations. Transit time was estimated for each of the ten ROIs. The parameters decay, epsilon, α, and β were global parameters.

In total, there are eight models specified and listed in Table 3.

#### 2.5.4. Two-way ANOVA analysis

A two-way ANOVA analysis was used to estimate the significant changes in the EC of individual ROIs and the connections between them. The change was calculated by subtracting the T6 EC values from the T5 EC values, as generated by DCM. The resulting values were then analyzed using Statistical Package for Social Sciences (SPSS). The dependent variables in ANOVA analysis were all the ROIs and connections between them, where sex and age at T5 were added as covariates, and the TOD group and congruence of image acquisition between T5 and T6 were fixed factors, i.e., independent variables in the ANOVA analysis.

The same procedure was applied for analyzing the parameters defining the BOLD signal, namely, decay and epsilon, which were global parameters as well as the BOLD signal per each ROI. The change between T5 and T6 in the mentioned parameters was calculated by subtracting T6 values from the T5 values as generated by DCM. The dependent variables in ANOVA analysis were averaged BOLD signal per each ROI, and two of the parameters defining the BOLD signal, namely, decay and epsilon. Age at T5 and gender were added as covariates, whereas the TOD group and congruence were fixed factors, i.e., independent variables in the ANOVA analysis.

## 3. Results

### 3.1. Effective connectivity

The results from PEB indicate that the model with the best fit to the data for effective connectivity parameters was model no. 8, which is the null model, at the posterior probability level of >0.95.

### 3.2. Parameters defining the BOLD signal

The results for the parameters defining the BOLD signal, namely, *transit time, epsilon, decay*, indicate that the overall winning model was model no. 4, time of day and sex. Models no. 2, sex; no. 7, time of day, sex, and age; and no. 6, sex and age exhibit a weak model fit with the data. The null model yielded no model evidence with the data.

### 3.3. ANOVA

The results from EC two-way ANOVA indicated that there were three significant connections:

- LIPC to right anterior PFC, *F* (5) = 2.705, *p* = 0.028
- Right anterior parietal to mPFC, *F* (5) = 3.188, *p* = 0.012
- Right anterior parietal (to itself), *F* (5) = 2.429, *p* = 0.044

There were no significant results from the parameters defining the BOLD signal, specifically global parameters of decay and epsilon, and BOLD signal parameters for each ROI.

## 4. Discussion

The findings from the current study suggest that the predefined models, namely age, time of day, and sex, and any combination of the three do not have a significant influence on the longitudinal EC data, as suggested by the PEB results. However, there are effects on the parameters that define the BOLD signal, transit time, epsilon, and decay, as the Bayesian model comparison indicated that PEB models including time of day and sex show the highest model evidence. This indicates that the specific combination of time of day and sex in this case exhibits a mirroring variability with the fMRI time series. These findings are in line with our previous study, in which we found no EC change throughout the day; however, we did report significant variation in the parameters defining the BOLD signal (14). We speculate that this is due to cardiovascular factors being more sensitive to circadian mechanisms in comparison to neural dynamics (28). While previous studies report a significant correlation between the regional blood flow and FC measures and their variability from morning to evening (29), this is only in part supported by our findings, where the parameters that define the BOLD signal exhibit variability based on the time of day. We largely attribute this contrast due to methodological differences, i.e., DCM vs. seed-based connectivity analysis of a previous publication.

Levels of hemoglobin have been shown to affect brain connectivity, differential for gender. We believe that these findings can be reflected in our findings on the parameters that define the BOLD signal, i.e., time of day and gender model (30).

The results from the ANOVA analysis indicated that in terms of change between the data collection waves, there were no significant group differences in the parameters that define the BOLD signal. There were, however, few significant EC connections, each ROI to itself and ROI to ROI (10^*^10), between the nodes of the fronto-parietal network and DMN, specifically the right anterior parietal network (fronto-parietal network) to itself and the mPFC (DMN) and RIPC (DMN) to the left anterior PFC (fronto-parietal network). A recent study that found variability in resting-state networks was affected by blood pressure, levels of hemoglobin, and hematocrit (13), mostly so in the DMN and its connections to other networks. The current publication has found that the EC in nodes of DMN and the fronto-parietal networks exhibit a significant change in connections between the test waves based on the time-of-day group and congruence between scanning times. While the current and mentioned study investigated different factors that may contribute to variation in resting-state networks, it can be concluded that DMN itself and its connectivity to other networks appear to be susceptible to endogenous and exogenous factors. These findings are in partial agreement with previously reported changes in the healthy elderly population, where the fronto-parietal network and SN exhibit changes in over 4 years (11); however, there were no significant changes in the DMN.

While it is difficult to directly compare and contrast with the majority of previously published studies due to methodological differences, specifically how FC is inferred from the fMRI time series using the mainstream analyses, i.e., correlating voxel intensities over time, where voxel intensities rely on the levels of oxy- and deoxyhemoglobin, we believe that our findings are complementary to previously reported findings on time of day variability in FC and low-frequency fluctuations (15, 19). Provided that we do observe variability in the parameters that define the BOLD signal, which can be explained by time of day and gender, we conclude that these factors should be accounted for in resting-state analyses.

Previous studies have indicated that time of day affects cognitive performance and resting-state network dynamics in fMRI paradigms in older adults, while the same results are not observed in younger individuals (31). The authors, therefore, suggest that the time of image acquisition in fMRI studies, especially in older adults, should be noted (31). Due to the restricted timespans in the current publication, the age of the participants was not strictly limited, i.e., the age span for the participants varied from 25 to 80 years of age; however, we have not observed age-dependent variability in neither EC nor the parameters defining the BOLD signal. It is plausible that broad age distribution and the small number of data points per each age in the current sample may have hindered the possible age effects in EC and parameters that define the BOLD signal.

In terms of brain morphology, the majority of findings consistently report brain volume changes throughout the day (16–18). This has been shown not to be the case in the longitudinal sample, where the authors of the publication report a minimal influence of the time of day, blood pressure, hydration, and caffeine intake on brain morphometric measures (20). However, in terms of the time of day, these contradictory findings are explained by minimal differences in the scanning time of the mentioned study compared to previously published reports. A review of blood pressure levels and brain morphology indicates that high blood pressure can lead to reductions in brain volume (32, 33).

To the best of our knowledge, this is the first publication addressing the importance of time of image acquisition and its congruence in longitudinal data collection procedures. With these findings, we want to express our strong suggestion for controlling the time of image acquisition in future fMRI studies. More importantly, we believe that reporting the data acquisition time in research reports, for example in the Methods section, may decrease the variability observed in the reported findings.

The overarching limitation of the current study is that the data used for the analysis were not collected with the aim of assessing the circadian mechanisms in the aging population; therefore, the study sample was relatively small and broadly distributed age-wise, as a result of predefined image acquisition times. In addition, the afternoon group timespan had to be broader, in comparison with the morning timespan, in order to achieve an appropriate sample size.

While the current study did not include information about the individual circadian chronotype, there seems to be a lack of consensus in the literature on whether the effects observed in resting-state measures are time of day/time of image acquisition dependent or driven by the individual circadian rhythmicity. Future studies investigating the circadian/time of day effects, ideally should include biological measures, such as blood pressure and levels of hemoglobin, in addition to individual circadian profiles and aim for longitudinal within individual sample collection.

## Data availability statement

The original contributions presented in the study are included in the article/supplementary material, further inquiries can be directed to the corresponding author.

## Ethics statement

This publication is based on data collected by the Betula prospective cohort study, Umeå University, Sweden, which involved human participants. All the patients/participants provided their written informed consent to participate in this study.

## Author contributions

LV and KS analyzed the data. LV prepared the manuscript. KS and AS supervised the methodological and theoretical aspects of the study. MA provided technical support and access to data. KS reviewed and approved the manuscript. All authors contributed to the article and approved the submitted version.
